# The role of grass volatiles on oviposition site selection by *Anopheles arabiensis* and *Anopheles coluzzii*

**DOI:** 10.1186/s12936-017-1717-z

**Published:** 2017-02-07

**Authors:** Yelfwagash Asmare, Sharon R. Hill, Richard J. Hopkins, Habte Tekie, Rickard Ignell

**Affiliations:** 1 Department of Zoological Sciences, Addis Ababa University, PO. Box 1176, Addis Ababa, Ethiopia; 2 Department of Biological Sciences, Debre Markos University, Debre Markos, Ethiopia; 3 Department of Plant Protection Biology, Unit of Chemical Ecology, Swedish University of Agricultural Sciences, Alnarp, Sweden; 4 Natural Resources Institute, University of Greenwich, London, UK

**Keywords:** *Anopheles arabiensis*, *Anopheles coluzzii*, Oviposition, Habitat, Selection, Olfaction, Attraction

## Abstract

**Background:**

The reproductive success and population dynamics, of *Anopheles* malaria mosquitoes is strongly influenced by the oviposition site selection of gravid females. Mosquitoes select oviposition sites at different spatial scales, starting with selecting a habitat in which to search. This study utilizes the association of larval abundance in the field with natural breeding habitats, dominated by various types of wild grasses, as a proxy for oviposition site selection by gravid mosquitoes. Moreover, the role of olfactory cues emanating from these habitats in the attraction and oviposition stimulation of females was analysed.

**Methods:**

The density of *Anopheles* larvae in breeding sites associated with *Echinochloa pyramidalis, Echinochloa stagnina, Typha latifolia* and *Cyperus papyrus*, was sampled and the larvae identified to species level. Headspace volatile extracts of the grasses were collected and used to assess behavioural attraction and oviposition stimulation of gravid *Anopheles arabiensis* and *Anopheles coluzzii* mosquitoes in wind tunnel and two-choice oviposition assays, respectively. The ability of the mosquitoes to differentiate among the grass volatile extracts was tested in multi-choice tent assays.

**Results:**

*Anopheles arabiensis* larvae were the most abundant species found in the various grass-associated habitats. The larval densities described a hierarchical distribution, with Poaceae (*Echinochloa pyramidalis* and *Echinochloa stagnina*)-associated habitat sites demonstrating higher densities than that of *Typha*-associated sites, and where larvae were absent from *Cyperus*-associated sites. This hierarchy was maintained by gravid *An. arabiensis* and *An. coluzzii* mosquitoes in attraction, oviposition and multi-choice assays to grass volatile extracts.

**Conclusions:**

The demonstrated hierarchical preference of gravid *An. coluzzii* and *An. arabiensis* for grass volatiles indicates that vegetation cues associated with larval habitats are instrumental in the oviposition site choice of the malaria mosquitoes. Identifying volatile cues from grasses that modulate gravid malaria mosquito behaviours has distinct potential for the development of tools to be used in future monitoring and control methods.

## Background

Oviposition site selection by gravid *Anopheles* malaria mosquitoes is a key moment in the reproductive success of the individual, and thus the population dynamics of the species [1, 2]. Consequently, the search for an oviposition site has important implications with regard to the control of malaria vectors [2]. When insects select oviposition sites, they make choices on increasingly fine spatial scales, starting with selecting a habitat in which to search [3–5]. In the case of egg-laying mosquitoes, they may have to leave a habitat in which they have been acquiring blood meals, in order to enter a habitat containing egg-laying sites. These habitats differ in physical, chemical and biological characteristics providing cues for the searching female, directly influencing the distribution, survival and abundance of mosquito larval populations [6–10]. The usage of an oviposition site by mosquitoes is dependent on their relative position in the landscape [8, 11], visual cues [12, 13], water vapour plumes [14], semiochemical cues associated with water bodies [15–18] and the physical parameters of the water [6, 7].

The most productive natural larval habitat types for *Anopheles gambiae/Anopheles coluzzii* and *Anopheles arabiensis* are transient puddles [1], often surrounded by short grasses [7, 9, 19, 20]. Both of these major vectors in sub-Saharan Africa have also been recorded in more stable water bodies, such as the littoral zone of lakes and in swamps [1, 19, 21–23]. Vegetation often populates these wetland habitats [1, 19, 23], and *An. gambiae/An. coluzzii* and *An. arabiensis* are commonly found amongst cattails (*Typha* spp.; Typhaceae) and dallis grasses (*Paspalum* spp.; Poaceae) [24–26]. In contrast, habitats populated by reeds (*Phragmites* spp.; Poaceae) and papyrus (*Cyperus papyrus*; Cyperaceae) generally produce low numbers of mosquitoes [19, 27, 28], probably due to the natural oil production of these species that reduces larval survivorship [29, 30]. Hence, grasses appear to play an important role in the natural breeding site selection of *An. arabiensis* and *An. gambiae/An. coluzzii*. Yet, the influence of natural grasses and other emergent vegetation on the oviposition site selection by gravid female *Anopheles* mosquitoes is not clearly understood. Moreover, the nature of the volatiles emitted from wild grasses and how they affect the behaviour of *An. arabiensis* and *An. gambiae/An. coluzzii* has not been investigated to date.

The objective of this study was to investigate anopheline larval occurrence and abundance in natural breeding habitats populated by four wild grass species: antelope grass, *Echinochloa pyramidalis* (Poaceae); hippo grass, *Echinochloa stagnina* (Poaceae); common cattail; *Typha latifolia* (Typhaceae); and papyrus reed, *C. papyrus* (Cyperaceae), and to correlate the behavioural response of gravid *An. arabiensis* and *An. coluzzii* to the natural volatiles collected from these grasses. The implications for anopheline ecology and vector management are discussed.

## Methods

### *Anopheles* larval density in habitats with emergent grass species

#### Study sites and sampling procedure

*Anopheles* larval sampling was made in potential breeding habitats at the southern littoral region of, and wetlands adjacent to, Lake Tana, Ethiopia (11°37′N, 37°21′E; 1830 m above sea level). The climate of the study area is typical of semi-arid regions close to the equator, with a high diurnal temperature variation between daytime extremes of 30 °C to night time lows of 6 °C, but mainly varies between 20 and 27 °C. Rainfall is on average 1440 mm per year, falling in one rainy season from May to October, with a peak during July–August [31]. During the El Niño event of 2014–2015, this region experienced a severe drought, with an overall reduction in rainfall of on average 50% [32], which had a drastic effect on *Anopheles* mosquito populations. For this reason, larvae of all stages, rather than first instars alone, were collected once in early September and again in late September, in an attempt to sample during the main proliferation period of mosquitoes in the study area.

*Cyperus papyrus* and *T. latifolia* are among the dominant grasses in deep water bodies of the lakeshore, whereas *E. pyramidalis* and *E. stagnina* predominantly are found at the edge of the lakeshore or in wetlands adjacent to the lake. In the study area, 10 sub-sites dominated by each individual grass species were selected. In each sub-site, 10 separate samplings of larvae (technical replicates) were made using a standard 350 ml dipper [33]. The collected *Anopheles* larvae were counted and recorded for each larval habitat associated with the different grass species. Of the collected larvae, 10% were preserved in 70% ethanol for subsequent identification to species using standard PCR analysis [34].

#### Data analysis

The data from the larval survey were subjected to a univariate general linear model (GLM), using the statistical software IBM SPSS Statistics for Windows, Version 21.0. Significant differences between means were determined at α = 0.05 and post hoc multiple comparisons among the grasses were made using the Tukey’s HSD test.

### Behavioural response of gravid mosquitoes to grass volatiles

#### Headspace odour collection

Freshly cut grass (100 g), including the vegetative and reproductive parts, was enclosed in a Teflon bag (Toppits, Cofresco, Germany). A charcoal-filtered continuous airstream (1 l min^−1^) was drawn by a Personal Air Sampler (PAS-500, Spectrex, Redwood City, CA, USA) over the grass onto an aeration column for 2 h. Aeration columns were made of Teflon tubing (4.5 cm × 3 mm i.d.), holding 50 mg Porapak Q (50/80 mesh; Waters Associates, Milford, MA, USA) between glass wool plugs. The columns were rinsed with 1 ml re-distilled *n*-hexane (Merck, Darmstadt, Germany) before use. Adsorbed volatiles were eluted with 300 µl re-distilled *n*-hexane. Odour collections from each grass species were pooled separately and then stored in sealed glass capillary tubes at −20 °C until used for behavioural experiments.

#### Mosquito rearing

*Anopheles arabiensis* (Dongola strain) and *An. coluzzii* (Suakoko strain) were kept at 27 ± 1 °C, 70 ± 5% RH, and at a 12 h:12 h light:dark photoperiod. Larvae were reared in plastic trays (22 cm × 34 cm × 10 cm) filled with 1 l distilled water, and fed powdered Tetramin^®^ fish food (Tetrawerke, Melle, Germany) daily. Pupae (80–100) were placed in BugDorm-1 insect cages (30 cm × 30 cm × 30 cm; Mega View Science, Taiwan) for adult emergence. Adult males and females were kept together and provided ad libitum access to 10% sucrose solution. For colony maintenance, female mosquitoes were blood fed on de-fibrinated sheep blood via a membrane-feeding system (Discover Workshops, Accrington, UK). Eggs were laid in 30 ml plastic cups (Nolato Hertila, Sweden) filled with distilled water, and then transferred to larval trays for hatching. For experiments, female mosquitoes, 4 days post-emergence, were allowed access to sheep blood (Håtunalab, Bro, Sweden) from an artificial feeder (Hemotek Discovery Workshops, Accrington, UK) for 3 h. Engorged females 6–8 h post-blood meal were then transferred to a new cage until used for experiments.

#### Wind tunnel bioassay

Attraction of *An. arabiensis* and *An. coluzzii* to the headspace odour extracts of the four grass species was analysed in a wind tunnel assay [35]. Cotton rolls (DAB Dental AB, Upplands Väsby, Sweden) were used as dispensers, and the amount of extract pipetted onto the dispensers corresponded to the amount of volatiles released during 0.04, 0.4, 4, 10 and 20 min from the individual grass species. An equivalent amount of *n*-hexane was used as a control. Both treatment and control dispensers were suspended from a 5 cm wire coil at the upwind end of the wind tunnel. Ten individual female mosquitoes, 48 h post-blood meal, were transferred to a release cage 2 h prior to experiments. The chambers were then placed in the downwind end of the wind tunnel, where the insects were allowed 5 min to adapt, before the butterfly valve of the cage was opened for their release. Attraction to either treatment or control was analysed as the proportion of mosquitoes that made source contact within 1 min after release. Each release rate for each grass volatile extract and the control was replicated ten times.

#### Oviposition bioassay

The oviposition response of *An. arabiensis* and *An. coluzzii* to the volatile extracts of the four grass species was analysed in BugDorm-1 insect cages kept in a climate-controlled room at 25 °C, 70 ± 5% RH, and at a 12 h:12 h light:dark photoperiod. Plastic cups (30 ml; Nolato Hertila) filled with 10 ml distilled water provided the oviposition substrate, and were located in opposite corners of the cage, 2 cm from each wall. The treatment cups were conditioned with one of the wild grass volatile extracts, in the same amounts used in the wind tunnel bioassay. An equivalent amount of *n*-hexane was used as a control. Treatment and control cups were exchanged in between each experiment. Ten mosquitoes, 48 h post-blood meal, were released into the experimental cages at 08:00–10:00, and the number of eggs in the treatment and control cups counted after 48 h. An oviposition index was determined by: (number of eggs laid in treatment cup − number of eggs laid in control cup)/(total number of eggs within the experimental cage). Each release rate of each grass volatile extract was replicated 5 times.

#### Tent bioassay

Greenhouse cage tents (2 m × 2 m × 2 m; BioQuip, Rancho Domniguez, CA, USA) were used as multi-array bioassays to analyse the oviposition preference of *An. arabiensis* and *An. coluzzii* to the four wild grass volatile extracts and a control. The tents were kept in a greenhouse at 27 ± 1 °C, 50 ± 5% RH, and at a 12 h:12 h light:dark photoperiod. As above, 30 ml plastic cups filled with 10 ml distilled water provided the oviposition substrate. Treatment cups were conditioned with the four wild grass volatile extracts in an amount corresponding to the volatiles released during 0.4 min from the individual grass species, while the control cup was conditioned with the equivalent amount of *n*-hexane. Treatment and control cups were set up in a 5 × 5 matrix (20 cm between cups). The matrix was changed in between replicates (n = 10 for each *Anopheles* species) to avoid position effects of the treatments. Twenty female mosquitoes, 48 h post-blood fed, were released into the tents, and the number of eggs counted after 24 h. The oviposition response was scored by counting the number of eggs in the treatment and control cups.

### Data analysis

The behavioural responses of gravid *An. coluzzii* and *An. arabiensis* in the wind tunnel and two-choice oviposition bioassay were analysed using a nominal logistic fit model, in which choice was the dependent variable, weighted by the number of (1) mosquitoes in the attraction assays and (2) eggs laid in the oviposition assays, with dose as the independent fixed effect and replicate as a random effect (JMP^®^ Pro 12.0.1. SAS Institute Inc., Cary, NC, USA). In the tent experiments (5-choice oviposition assays), the number of eggs was used as the weight, the choice as the dependent variable, the grasses and control as the independent fixed effect, and the tent and replicates as the random effects. The χ^2^ and *P* value from the likelihood ratio test are reported here. Significant differences between the individual doses (wind tunnel and two-choice assays) and grasses (five-choice assay) were determined by odds ratio pairwise comparisons.

## Results

### *Anopheles arabiensis* larval density in natural grass habitats

No significant effect of sub-site location was found (*F* = 0.367, *DF*_*d*_ = 9, *DF*_*n*_ = 3, *P* = 0.999). Hence, the larval abundance data collected from the breeding habitats associated with each grass species was pooled in subsequent analysis. *Anopheles arabiensis* was the most abundant species comprising more than 40% of the specimens in the study area, and was the only member of the *An. gambiae* sensu lato complex to be identified following PCR analyses of 48 mosquito larvae.

**Fig. 1:**
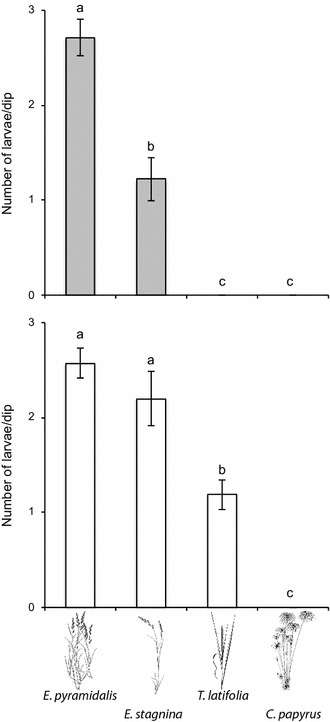
*Anopheles* larval density in natural breeding habitats dominated by four grass species, *Echinochloa pyramidalis*, *Echinochloa stagnina*, *Typha latifolia* and *Cyperus papyrus*, assayed in early (*top*) and late (*bottom*) September. The mean larval densities with different letter designations are significantly different from one another (univariate general linear model with a Tukey’s post hoc analysis; *P* < 0.005). *Error bars* represent the standard error of the mean

A significantly higher number of *An. arabiensis* larvae were found in *E. pyramidalis* dominated breeding habitats than in any of the other potential breeding habitats (Fig. 1). The number of larvae in the remaining habitats also differed significantly, with breeding habitats dominated by *E. stagnina* containing more larvae than breeding habitats dominated by *T. latifolia* (Fig. 1). Interestingly, no larvae were found in habitats dominated by *C. papyrus* (Fig. 1).

### Attraction of *An. arabiensis* and *An. coluzzii* to grass volatiles

**Fig. 2:**
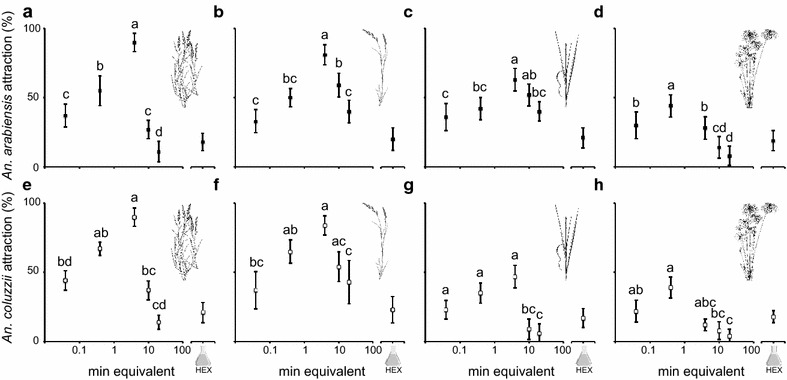
Attraction of gravid *Anopheles arabiensis* and *Anopheles coluzzii* to grass volatile headspace extracts, from *Echinochloa pyramidalis* (**a**, **e**) *Echinochloa stagnina* (**b**, **f**), *Typha latifolia* (**c**, **g**) and *Cyperus papyrus* (**d**, **h**), respectively. Release rate of the volatile headspace extracts is given in minute equivalents. The solvent control was hexane (HEX). The mean percent attraction values with the* same letters* indicate no significant difference from one another (nominal logistic fit model, χ^2^ and *P* < 0.05 from the likelihood ratio test, significant differences were determined by odds ratio pairwise comparisons). *Error bars* represent the standard error of the mean

Attraction of *An. arabiensis* and *An. coluzzii* to the grass volatile extracts was dose dependent and differed among the grass species (*An. arabiensis*: Dose, χ^2^ = 34.51, *P* < 0.0001; Grass, χ^2^ = 13.11, *P* = 0.0044; *An. coluzzii*: Dose, χ^2^ = 46.25, *P* < 0.0001; Grass, χ^2^ = 41.46, *P* < 0.0001) (Fig. 2). No significant difference in the attraction of *An. arabiensis* to the volatile extracts of *E. pyramidalis, E. stagnina* and *T. latifolia* was found, however, the attraction to the *C. papyrus* volatile extract was significantly lower than each of the other grass volatile extracts (Table 1). Attraction of *An. coluzzii* to the volatile extracts of either the Poaceae (*E. pyramidalis* and *E. stagnina*), or the Typhaceae (*T. latifolia*) and Cyperaceae (*C. papyrus*), did not differ. The attraction to the volatile extract of the Typhaceae and Cyperaceae, however, was significantly lower than that of the Poaceae (Table 1).

**Table 1:** Cross-comparative analysis of the attraction of gravid mosquitoes to volatile extracts of grass species

		Attraction			
		*An. arabiensis*		*An. coluzzii*	
Level 1	Level 2	Odds ratio	*P*	Odds ratio	*P*
*E. pyramidalis*	*E. stagnina*	0.8361	0.3210	0.8929	0.5313
*E. pyramidalis*	*T. latifolia*	0.9226	0.6546	2.011	0.0002
*E. pyramidalis*	*C. papyrus*	1.553	0.0181	2.431	<0.0001
*E. stagnina*	*T. latifolia*	1.103	0.5802	2.252	<0.0001
*E. stagnina*	*C. papyrus*	1.858	0.0007	2.722	<0.0001
*T. latifolia*	*C. papyrus*	1.684	0.0045	1.209	0.3436

The analysis is done using a nominal logistic regression model fit

α = 0.05

### Oviposition response of *An. arabiensis* and *An. coluzzii* to grass volatiles

**Fig. 3:**
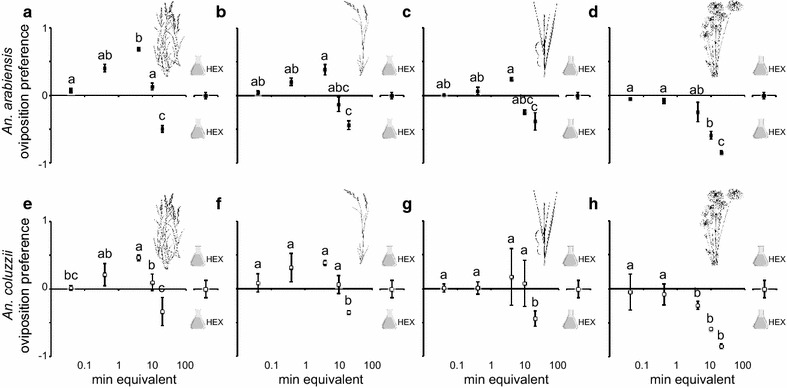
Oviposition response of gravid *Anopheles arabiensis* and *Anopheles coluzzii* to grass volatile headspace extracts, from *Echinochloa pyramidalis* (**a**, **e**) *Echinochloa stagnina* (**b**, **f**), *Typha latifolia* (**c**, **g**) and *Cyperus papyrus* (**d**, **h**), respectively. Release rate of the volatile headspace extracts is given in minute equivalents. The solvent control was hexane (HEX). Mean oviposition preferences with the *same letters* indicate no significant difference from one another (nominal logistic fit model, χ^2^ and *P* < 0.05 from the likelihood ratio test, significant differences were determined by odds ratio pairwise comparisons). *Error bars* represent the standard error of the mean

The oviposition response of *An. arabiensis* and *An. coluzzii* to water conditioned with the grass volatile extracts was both dose dependent and differed among the grass species (*An. arabiensis*: Dose, χ^2^ = 94.67, *P* < 0.0001; Grass, χ^2^ = 44.79, *P* < 0.0001; *An. coluzzii*: Dose, χ^2^ = 47.35, *P* < 0.0001; Grass, χ^2^ = 24.29, *P* < 0.0001) (Fig. 3). No significant differences in oviposition response were found for *An. coluzzii* to water conditioned with volatile extracts of *E. pyramidalis, E. stagnina* and *T. latifolia,* but the oviposition response to these grass extracts were significantly higher than that observed for the *C. papyrus* volatile extract (Table 2). Similarly, for the *C. papyrus* volatile extract, the oviposition response of *An. arabiensis* was significantly lower than to that of all the other grass volatile extracts (Table 2). In contrast to *An. coluzzii*, the volatile extract of *T. latifolia* stimulated a lower oviposition response in *An. arabiensis* than that of *E. pyramidalis* (Table 2).

**Table 2:** Cross-comparative analysis of the oviposition response of gravid mosquitoes to volatile extracts of grass species

		Oviposition			
		*An. arabiensis*		*An. coluzzii*	
Level 1	Level 2	Odds ratio	*P*	Odds ratio	*P*
*E. pyramidalis*	*E. stagnina*	0.8425	0.0687	1.005	0.9556
*E. pyramidalis*	*T. latifolia*	0.7780	0.0076	0.8700	0.1261
*E. pyramidalis*	*C. papyrus*	0.5364	<0.0001	0.6800	<0.0001
*E. stagnina*	*T. latifolia*	0.9233	0.3932	0.8657	0.1043
*E. stagnina*	*C. papyrus*	0.6366	<0.0001	0.6766	<0.0001
*T. latifolia*	*C. papyrus*	0.6894	<0.0001	0.7816	0.0067

The analysis is done using a nominal logistic regression model fit

α = 0.05

### Tent experiments response

**Fig. 4:**
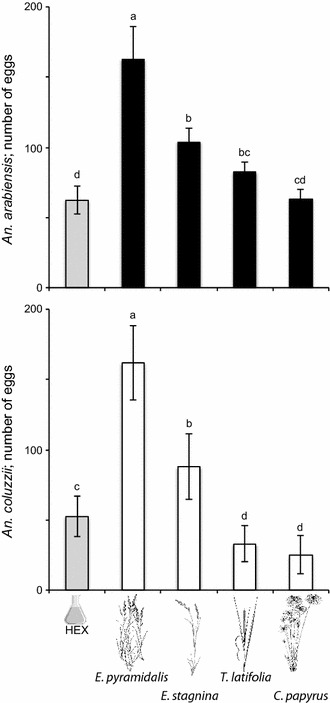
Oviposition preference hierarchy of *Anopheles arabiensis* (*top*) and *Anopheles coluzzii* (*bottom*) in a multi-choice arena, to water conditioned with volatile headspace extracts of the four grass species, *Echinochloa pyramidalis*, *Echinochloa stagnina*, *Typha latifolia* and *Cyperus papyrus*. Mean numbers of eggs with the same letters indicate no significant difference from one another (nominal logistic fit model, χ^2^ and *P* < 0.05 from the likelihood ratio test, significant differences were determined by odds ratio pairwise comparisons). *Error bars* represent the standard error of the mean

In the five-choice oviposition assay, the overall oviposition preference hierarchy of both *Anopheles* species to the grass volatile extracts was found to be *E. pyramidalis* > *E. stagnina* ≥ *T. latifolia* = *C. papyrus* (Fig. 4). Water conditioned with volatile extracts of either *E. pyramidalis* or *E. stagnina*, elicited a significantly higher oviposition response than the control in both *An. arabiensis* and *An. coluzzii,* while that of *T. latifolia* elicited a significantly higher response than the control in *An. arabiensis* alone (Fig. 4). In contrast, the oviposition response of *An. coluzzii* to water conditioned with volatile extracts of *T. latifolia,* as well as that of *C. papyrus*, was significantly lower than that of the control.

## Discussion

Field collection of *An. arabiensis* larvae indicated a role for emergent vegetation in the oviposition site selection and survival of malaria mosquitoes. Larval densities were highest in Poaceae-associated habitats, much lower in *Typha*-associated sites and absent from *Cyperus*-associated sites. One potential mechanism regulating the differential distribution of larvae may be an odour-based oviposition site selection preference. Gravid female *An. arabiensis* and *An. coluzzii* were, indeed, found to be differentially attracted to all grass volatile extracts, yet were only stimulated to oviposit on water conditioned with Poaceae volatile extracts. This was further supported by multi-choice oviposition assays revealing that both species demonstrated a preference hierarchy among the grass volatiles, *E. pyramidalis* > *E. stagnina* > *T. latifolia* ≥ *C. papyrus*. These findings may also reflect the abundance of available nutrients and toxins associated with these potential larval habitats, which may affect larval survival differentially in the various grass-associated habitats.

Emergent vegetation in aquatic habitats are commonly associated with the presence or absence of *An. gambiae/An. coluzzii* and *An. arabiensis* larvae [1, 7, 9, 19, 23, 36]. While the number of *Anopheles* larvae collected in this study were low, due to the impact of El Niño in the study area in 2014–2015, the observed patterns of association with vegetation are consistent with previous reports [9, 19, 24]. In studies in which the vegetation has been characterised, habitats associated with Poaceae generally have a higher *An. gambiae/An. coluzzii* and *An. arabiensis* larval density compared with that of habitats associated with Typhaceae and Cyperaceae [9, 19, 24]. While vegetation in these habitats is known to influence characteristics, such as shading, temperature, water flow, predator abundance and nutrients, they also provide gravid mosquitoes with chemical cues important to habitat selection.

An increasing body of evidence suggests that anopheline mosquitoes make use of olfactory cues as positive indicators for oviposition site selection [18, 37–39]. While the focus of this study was not to identify the specific salient volatiles in these attractive and aversive grasses, the behavioural results presented here indicate a strong and robust preference for the headspace extracts of the Poaceae grasses. Interestingly, the majority of the previously identified olfactory cues that drive the oviposition site selection in *An. gambiae s.l.* originate from wild and cultivated grasses of the Poaceae family [37–39]. These odours include α- and ß-pinene, 3-carene, caryophyllene, limonene and nonanal [18, 38–41], which are not affected by mechanical damage of the plants, but are thought to be constitutively expressed [42–44]. Future work will be aimed at identifying the salient volatiles in the grasses that elicited attraction in gravid anophelines in this study.

*Anopheles* mosquitoes are likely differentially attracted to larval habitats that are rich in nutrients, derived directly from shed pollen [45–47] and indirectly from accumulated detritus and associated micro-organisms, and that provide shelter from abiotic and biotic threats [48]. Nutrients derived from shed maize pollen, which are rich in water soluble proteins [49], have been shown to enhance larval development and growth, adult size and survival [45, 46]. While pollen of other grass species might also constitute a supplemental nutriment for surface feeding anophelines, the nutritive quality of grass pollen varies across species [50, 51]. For example, typha pollen is known to be nutrient poor compared with maize pollen [50, 51]. Females that select larval habitats associated with grasses shedding nutrient rich pollen have the potential to increase their own fitness by providing their offspring with selective advantages, as previously shown for maize pollen. Selective pressures may also be involved in the avoidance of *C. papyrus*-associated habitats by gravid anophelines, as this grass secretes essential oils that create a thin film on the surface of the water, preventing mosquito larvae from breathing [29, 30].

## Conclusions

The demonstrated hierarchical preference of gravid *An. coluzzii* and *An. arabiensis* for grasses indicates that vegetation types associated with larval habitats are instrumental in the oviposition choice of the malaria mosquitoes. Current and future analysis of *Anopheles* oviposition ecology associated with grasses is likely to provide key larval habitat characteristics that may be integrated into current methods of larval control. Also, identifying volatile cues from grasses that modulate gravid malaria mosquito behaviours has distinct potential for the development of tools to be used in future monitoring and control methods.

## Abbreviations

DF: degrees of freedom
GLM: generalised linear model
HSD: honest significant difference
PCR: polymerase chain reaction
RH: relative humidity
χ^2^: Chi squared
